# Genome-Wide Characterization of Phosphate Transporters Reveals Differential Regulation of Growth and Medicinal Metabolite Accumulation Under Phosphorus Deficiency in *Ginkgo biloba*

**DOI:** 10.3390/biology15191676

**Published:** 2026-09-22

**Authors:** Yadi Chen, Chi Zhang, Fangmei Cheng, Qingtao Xu, Weixing Li

**Affiliations:** 1 College of Horticulture and Landscape Architecture, Yangzhou University, Yangzhou 225009, China; yadichen@yzu.edu.cn (Y.C.); mz120241556@stu.yzu.edu.cn (C.Z.); mx120240866@stu.yzu.edu.cn (Q.X.); 2 Jiangsu Coastal Area Institute of Agricultural Sciences, Yancheng 224002, China; chengfangmei@126.com

**Keywords:** *Ginkgo biloba*, phosphorus deficiency, flavonoids, secondary metabolism, phosphate transporter

## Abstract

*Ginkgo biloba* L. (*G. biloba*) is an important medicinal tree species rich in bioactive flavonoids and terpene lactones. Phosphate transporters (PHTs) play essential roles in phosphate uptake, transport, and homeostasis; however, their characteristics and functions in *G. biloba* remain unclear. In this study, 21 non-redundant GbPHT genes were identified from the *G. biloba* genome and classified into five subfamilies, including PHT1, PHT2, PHT3, PHT4, and PHO, containing five, one, four, six, and five members, respectively. Promoter analysis revealed abundant cis-elements associated with light, hormone, and stress responses, suggesting complex regulatory patterns of *GbPHT* genes. To investigate the effects of phosphorus availability on growth and medicinal metabolites, *G. biloba* seedlings were subjected to normal phosphorus and phosphorus deficiency treatments. Phosphorus deficiency significantly inhibited root development, biomass accumulation, and photosynthetic performance. However, phosphorus deficiency markedly enhanced flavonoid accumulation in leaves and terpene lactone accumulation in roots, accompanied by increased expression of key structural genes involved in their biosynthetic pathways. Furthermore, transient overexpression of *GbPHT1-1* in *G. biloba* leaves promoted flavonoid accumulation and upregulated flavonoid biosynthetic genes. Collectively, this study provides the first comprehensive characterization of the *G. biloba* PHT family and reveals that phosphorus deficiency coordinates growth restriction with enhanced medicinal secondary metabolite accumulation. These findings provide new insights into phosphorus adaptation and nutrient regulation of medicinal quality in *G. biloba*.

## Simple Summary

*Ginkgo biloba* is an important medicinal tree valued for its bioactive flavonoids and terpene lactones. Phosphorus is essential for plant growth, but how its deficiency affects *Ginkgo* growth and medicinal quality remains poorly understood. In this study, we investigated the physiological, metabolic, and molecular responses of *Ginkgo* seedlings to phosphorus deficiency. Phosphorus deficiency strongly inhibited root development, biomass accumulation, and photosynthetic performance. In contrast, it promoted flavonoid accumulation in both leaves and roots and enhanced terpene lactones in roots, accompanied by the upregulated expression of key biosynthetic genes. Furthermore, 21 phosphate transporter genes were identified in the *Ginkgo* genome, showing divergent expression under phosphorus starvation. Among them, *GbPHT1-1* was markedly induced in leaves, and its transient overexpression further stimulated flavonoid accumulation and biosynthetic gene expression. Overall, our findings demonstrate that phosphorus deficiency restricts growth while enhancing the accumulation of valuable secondary metabolites, providing new insights into phosphorus adaptation and medicinal quality regulation in *Ginkgo*.

## 1. Introduction

Phosphorus (P) is one of the essential macronutrients required for plant growth and development, participating in numerous biological processes including energy metabolism, nucleic acid synthesis, membrane formation, and signal transduction [1,2,3]. However, the availability of inorganic phosphate (Pi), the primary form absorbed by plants, is frequently limited in natural soils because of its low mobility and strong fixation by mineral components [4,5]. Consequently, phosphorus deficiency has become one of the major constraints limiting plant productivity worldwide [3,6]. Plants have evolved a series of adaptive strategies to cope with phosphorus-deficiency conditions, including remodeling of root architecture, enhancement of phosphorus acquisition and utilization efficiency, metabolic reprogramming, and extensive transcriptional regulation [7,8,9]. In addition to suppressing vegetative growth, increasing evidence indicates that phosphorus deficiency substantially alters plant metabolism, particularly the biosynthesis of secondary metabolites that contribute to environmental adaptation and stress tolerance [10]. Nevertheless, the molecular mechanisms coordinating phosphorus acquisition with metabolic adaptation remain incompletely understood.

Phosphate transporters (PHTs) play indispensable roles in maintaining phosphorus homeostasis by mediating Pi uptake, transport, intracellular redistribution, and storage [11,12]. Based on structural and subcellular characteristics, plant PHTs are classified into PHT1, PHT2, PHT3, PHT4, PHT5 and PHO subfamilies [13]. Among them, plasma membrane-localized PHT1 members function predominantly in high-affinity Pi uptake from the soil and represent the major components responding to phosphorus starvation [14,15]. While the transport functions of PHT proteins have been extensively characterized in model annuals and cereal crops with a focus on phosphate uptake, transport, and homeostasis [3,16,17,18], how the PHT family has evolved and functions in perennial gymnosperms with large and highly repetitive genomes remains poorly defined. Moreover, because phosphorus availability is closely associated with metabolic adjustment in plants, the potential connection between PHT-mediated phosphate transport and metabolic responses to phosphorus deficiency remains less explored, particularly with respect to the biosynthesis of specialized medicinal secondary metabolites in woody species.

*Ginkgo biloba* is a relict gymnosperm and one of the most economically important medicinal tree species worldwide [3,19]. Standardized *Ginkgo* leaf extracts are widely used for the prevention and treatment of cardiovascular and neurodegenerative diseases, and their pharmacological activities are primarily attributed to flavonoid glycosides and terpene lactones [20,21]. Therefore, maintaining or enhancing the accumulation of these bioactive compounds is of considerable importance for medicinal quality. Previous studies have shown that environmental factors, including light, temperature, water availability, and mineral nutrition, markedly influence the biosynthesis of flavonoids and terpene lactones in *Ginkgo* [22,23,24,25]. Nutrient availability is closely linked to carbon allocation between vegetative growth and secondary metabolite accumulation [26]. Although the regulatory mechanisms of phosphorus nutrition have been extensively investigated in model plants and crop species, how phosphorus deficiency affects growth and the accumulation of major medicinal metabolites in woody medicinal plants remains poorly understood. More importantly, whether and how PHTs participate in phosphorus-responsive metabolic responses in *G. biloba*, particularly in relation to flavonoid accumulation, has not been well characterized.

In the present study, we performed a genome-wide identification and characterization of the PHT gene family in *G. biloba*, systematically analyzing their evolutionary relationships, gene structures, conserved motifs, promoter cis-elements, and expression patterns under phosphorus deficiency. This genome-wide analysis enabled us to characterize the PHT family in *G. biloba* and identify candidate members responsive to phosphorus deficiency. We then investigated the physiological consequences of low-phosphorus stress on seedling growth, photosynthetic performance, and the accumulation dynamics of flavonoids in leaves and terpene lactones in roots. Comprehensive qRT-PCR expression profiling under phosphorus- deficiency conditions further identified *GbPHT1-1* as a primary candidate strongly induced by phosphorus deficiency in leaves. To further assess its potential function, an *Agrobacterium*-mediated transient overexpression assay of *GbPHT1-1* was performed in ginkgo leaves, and its effects on flavonoid accumulation and the expression of structural biosynthetic genes were subsequently evaluated. Collectively, this study provides a comprehensive characterization of the *G. biloba* PHT family and provides evidence for a potential association between PHT and medicinal secondary metabolism under phosphorus deficiency, providing new insights into the molecular mechanisms underlying phosphorus adaptation in woody medicinal plants.

## 2. Materials and Methods

### 2.1. Identification of GbPHT Gene Family Members

The genomic sequences, protein sequences and annotation files of *G. biloba* (version-2019) were obtained from the GinkgoDB database (https://ginkgo.zju.edu.cn/), accessed on 29 September 2025. Twenty-three *Arabidopsis thaliana* (*A. thaliana*) phosphate transporter (AtPHT) protein sequences were downloaded from The *Arabidopsis* Information Resource (TAIR, https://www.arabidopsis.org/), accessed on 28 September 2025, and used as queries in bidirectional BLAST searches against the *G. biloba* genome with TBtools software (v2.012) to identify candidate homologous genes. The choice of candidate PHTs was based on an E value < 1 × 10^−5^ and a query coverage over 50%. All candidate phosphate transporter protein sequences were confirmed based on the conserved domain accession numbers PF00083 (PHT1), PF01384 (PHT2), PF00153 (PHT3), PF03124 (PHO) and PF03105 (PHO) in the Pfam database (https://www.ebi.ac.uk/interpro/entry/pfam/#table), accessed on 29 September 2025. The physicochemical properties of PHT proteins, including amino acid length, molecular weight (MW), theoretical isoelectric point (pI), instability index (II), aliphatic index and grand average of hydropathicity (GRAVY), were calculated using the ExPASy ProtParam server (https://web.expasy.org/protparam/), accessed on 1 July 2026. Subcellular localization was predicted with CELLO v.2.5 (https://cello.life.nctu.edu.tw/), accessed on 1 July 2026, and the number of transmembrane helices (TMHs) was determined with TMHMM Server v.2.0 (http://www.cbs.dtu.dk/services/TMHMM) via default parameters, accessed on 1 July 2026.

### 2.2. Chromosomal Localization and Phylogenetic Analysis of the GbPHT Gene Family

The annotated GFF file of the *G. biloba* genome was downloaded from GinkgoDB (https://ginkgo.zju.edu.cn/), accessed on 29 September 2025, and chromosome density distribution maps were generated with TBtools software (v2.012). The *A. thaliana* PHT protein sequences were obtained from the *Arabidopsis* Information Resource (TAIR, https://www.arabidopsis.org/), accessed on 28 September 2025. The *Populus trichocarpa* (*P. trichocarpa*) PHT protein sequences were obtained from the NCBI database (https://www.ncbi.nlm.nih.gov/), accessed on 2 July 2026. The rice PHT protein sequences were downloaded from the China Rice Data Center (https://www.ricedata.cn/), accessed on 29 September 2025. All PHT family members annotated in the respective databases were included in the analysis without further filtering. To investigate evolutionary relationships, multiple sequence alignment of PHT proteins from *A. thaliana*, *Oryza sativa* (*O. sativa*), *P. trichocarpa* and *G. biloba* was performed with MEGA 11 software with default parameters. A phylogenetic tree was constructed using the neighbor-joining method with 1000 bootstrap replicates and visualized using the Interactive Tree of Life (iTOL, https://itol.embl.de/), accessed on 6 July 2026.

### 2.3. Gene Structure, Conserved Motif and Domain Analyses

Conserved protein motifs were identified using MEME Suite (https://meme-suite.org/meme/tools/meme), accessed on 9 December 2025, with the maximum number of motifs set to 20. Conserved domains were predicted using the NCBI Conserved Domain Database (CDD). Gene exon-intron structures were extracted from the genome annotation file and visualized with TBtools.

### 2.4. Promoter cis-Element Analysis

The 2-kb genomic sequences upstream of the translation start codon (ATG) of each *GbPHT* gene were extracted using TBtools as promoter regions. Putative cis-acting regulatory elements were predicted using the PlantCARE database (http://bioinformatics.psb.ugent.be/webtools/plantcare/html/), accessed on 5 January 2026, and the results were visualized with TBtools [27].

### 2.5. Plant Materials and Phosphorus Treatments

*G. biloba* seeds were collected from the Ginkgo Experimental Station of Yangzhou University, Yangzhou, Jiangsu Province, China (32°39′ N, 119°43′ E), and stratified in moist perlite at 4 °C. Before sowing, seeds were pre-germinated in a growth chamber at 25 °C under a 16 h light/8 h dark photoperiod for 7 d. Uniformly germinated seeds were then sown in a mixture of nutrient soil and vermiculite at a 3:1 ratio (*v*/*v*) and grown in a controlled growth chamber at 25 °C during the light period and 18 °C during the dark period. Twenty-four uniform 19-day-old *G. biloba* seedlings with healthy root systems were selected for hydroponic culture. After the residual substrate was gently removed from the roots with deionized water, the seedlings were transferred to a modified half-strength Hoagland nutrient solution (Cat. No. NS1010-P; Coolaber Technology Co., Ltd., Beijing, China) containing either normal phosphorus (200 μmol·L^−1^ KH_2_PO_4_, 200 P) or phosphorus-deficient solution (0 μmol·L^−1^ KH_2_PO_4_, 0 P) [28,29]. The nutrient solution contained K^+^ supplied by KNO_3_ and KI, and was renewed every three days. All seedlings were grown in a controlled growth chamber at 25 °C under a 16 h light/8 h dark photoperiod. Each treatment included three biological replicates, with each replicate comprising four seedlings grown under identical conditions. The experimental unit was defined as an individual *G. biloba* seedling. After 34 days of treatment, plant growth parameters were recorded, and leaf and root samples were collected for subsequent physiological and molecular analyses.

### 2.6. Determination of Plant Growth Traits

Primary root length was measured using a digital caliper. Leaf area was determined from digital images using ImageJ software (v1.54g). Leaf fresh weight (FW) and dry weight (DW) were measured using an analytical balance after oven drying at 75 °C to constant weight.

### 2.7. Determination of Photosynthetic Pigments

Chlorophyll and carotenoid contents were determined according to the ethanol extraction method. Briefly, 0.10 g of fresh leaf tissue was extracted with 95% ethanol in darkness until complete decolorization. Absorbance was measured at 665, 649 and 470 nm using a UV-visible spectrophotometer. Chlorophyll a, chlorophyll b, total chlorophyll and carotenoid contents were calculated according to standard equations.

Chlorophyll a (Ca) = (13.95 × A665 − 6.8 × A649) × V/(1000 × W) (unit: mg/g)

Chlorophyll b (Cb) = (24.96 × A649 − 7.32 × A665) × V/(1000 × W) (unit: mg/g)

Carotenoid contents (Cc) = (1000 × A470 − 2.05 × Ca − 114.8 × Cb)/245 × V/(1000 × W) (unit: mg/g)

Total chlorophyll (Cr) = Chlorophyll a (Ca) + Chlorophyll b (Cb) (unit: mg/g)

where A represents the absorbance at the specified wavelength, V is the extraction volume (mL), and W is the fresh weight (g).

### 2.8. Measurement of Photosynthetic Parameters

Photosynthetic parameters of *G. biloba* leaves were measured using a portable photosynthesis system (Model 3051D, Zhejiang Top Cloud-Agri Technology Co., Ltd., Hangzhou, China). To ensure consistency in circadian time and light stability, all measurements were strictly conducted between ZT3 and ZT4 in the growth chamber. For each treatment, five fully expanded leaves at the same developmental position in the middle-upper canopy were selected from each of the 12 seedlings and measured. These five leaves per seedling were treated as technical replicates (repeated measurements) and averaged to obtain a single value per seedling before statistical analysis. During the measurements, the average photosynthetically active radiation (PAR) in the leaf chamber was 1029.575 µmol m^−2^ s^−1^. The average reference CO_2_ concentration was 277.07 ppm. The average air temperature in the leaf chamber was controlled at 26.67 °C (with an average leaf temperature of 26.25 °C), and the relative humidity was maintained at approximately 52.39%.

### 2.9. Chlorophyll Fluorescence Analysis

Chlorophyll fluorescence parameters were measured using a PlantView 230F chlorophyll fluorescence imaging system (Brolight, Hangzhou, China). Leaves were dark-adapted for 30 min prior to measurement. The maximum quantum efficiency of PSII (*Fv*/*Fm*) and fluorescence images were subsequently obtained and analyzed.

### 2.10. Determination of Flavonoid Content

Leaf and root samples were oven-dried, ground into fine powder, and sieved through an 80-mesh screen. Total flavonoid content was determined using a commercial Plant Flavonoid Assay Kit (Suzhou Comin Biotechnology Co., Ltd., Suzhou, China) according to the manufacturer’s instructions. Briefly, 0.02 g of dried powder was extracted with 2 mL of 60% ethanol at 60 °C for 2 h with oscillation (sample-to-solvent ratio: 1:100, *w*/*v*). The extract was centrifuged at 10,000 rpm for 10 min at 25 °C, and the supernatant was used for the assay. The absorbance of the reaction mixture was measured at 510 nm, zeroed with distilled water. Total flavonoid content was calculated using the formula provided by the manufacturer: Content (mg/g DW) = (ΔA − 0.0007)/5.02/(W/Vt), where ΔA is the difference in absorbance between the sample and the blank, W is the sample weight (g) and Vt is the total volume of the extraction solvent (2 mL). Three biological replicates were included for each treatment.

### 2.11. Determination of Terpene Lactones (HPLC)

Leaves were subjected to enzyme inactivation (blanching) and ground into a fine powder. Approximately 150.0 mg of the powdered sample was accurately weighed and transferred into a clean 2 mL centrifuge tube. Subsequently, 1.0–1.5 mL of methanol was added, and the mixture was thoroughly vortexed and sonicated for 30 min. After centrifugation at 12,000 rpm for 5 min, the supernatant was collected, and the extraction procedure was repeated twice. The three pooled supernatants were passed through an acidic alumina column and eluted with 5 mL methanol. The eluates were collected and evaporated to dryness under a gentle stream of nitrogen using a nitrogen evaporator in a fume hood. The dried residues were redissolved in 1.00 mL methanol, vortexed to completely wash the tube walls, and sonicated to facilitate dissolution. Subsequently, 1.00 mL ddH_2_O was added, and the mixture was vortexed thoroughly. After ultrasonic-assisted dissolution, 1 mL of the extract was filtered through a 0.22 μm membrane filter prior to high-performance liquid chromatography (HPLC) analysis. The contents of bilobalide, ginkgolide A, ginkgolide B, ginkgolide C, and total terpene lactones were determined using an Agilent Infinity II 1260 HPLC system equipped with an Agilent EC-C18 column (3.0 × 150 mm, 2.7 μm). The mobile phase consisted of 75% water and 25% methanol, at a flow rate of 0.5 mL/min, with a column temperature of 40 °C and an injection volume of 10 μL. Detection was carried out with a refractive index detector, and identification was based on the retention times of reference standards.

### 2.12. Determination of H_2_O_2_ Content and SOD Activity

The Hydrogen peroxide (H_2_O_2_) content and the activities of superoxide dismutase (SOD) were determined according to the manufacturer’s instructions via commercial assay kits (Suzhou Comin Biotechnology, Suzhou, China).

### 2.13. Agrobacterium-Mediated Transient Transformation

The coding sequence (CDS) of *GbPHT1-1* was amplified using an LA Taq Kit (Takara, Kusatsu, Japan) following the manufacturer’s protocols and using *G. biloba* cDNA as a template. The PCR products were subjected to agarose gel electrophoresis and purified using the Gel Extraction Kit (CoWin Biosciences, Beijing, China). The CDS of *GbPHT1-1* was cloned into the overexpression vector 35S::FGFP, and each was digested with BamHI, using a ClonExpress II One Step Cloning Kit (Vazyme Biotech, Nanjing, China). Plasmids from sequence-verified clones were extracted and transformed into *Agrobacterium tumefaciens* strain AGL1. Single colonies were inoculated into LB liquid medium containing 50 μg/mL kanamycin and 25 μg/mL rifampicin and cultured overnight at 28 °C with shaking at 220 rpm, reaching an OD_600_ of approximately 2.0. Bacterial cells were collected by centrifugation at 5000 rpm for 10 min at room temperature, and the supernatant was discarded. The pellet was resuspended in an infiltration medium consisting of 1/4 MS, 10 mM MgCl_2_, 1% (*w*/*v*) sucrose, 100 μM acetosyringone, and 0.01% (*v*/*v*) Silwet L-77, with the final OD_600_ adjusted to approximately 0.6. The suspension was injected into the abaxial side of leaves from 40-day-old *G. biloba* plants using a 1-mL needleless syringe. For each treatment (excluding mock-infiltrated plants), we infiltrated 15 independent *G. biloba* seedlings, and from each plant three fully expanded top leaves were infiltrated, giving 45 infiltrated leaves per treatment. Infiltrated plants were kept in darkness for 12 h and subsequently transferred to a growth chamber under controlled conditions. At 5 d post-infiltration, the injected leaves were harvested to quantify target gene expression levels and determine flavonoid content.

### 2.14. RNA Extraction and Quantitative Real-Time Polymerase Chain Reaction (qRT-PCR)

Total RNA was extracted using a FastPure Universal Plant Total RNA Isolation Kit (Vazyme, RC411). RNA concentration and purity were assessed using a NanoDrop One spectrophotometer (Thermo Fisher Scientific, Madison, WI, USA; manufactured in China). Only RNA samples with an A260/A280 ratio between 1.8 and 2.0 and an A260/230 ratio between 1.8 and 2.2 were used for subsequent experiments. RNA samples with genomic DNA contamination were treated with 4 × gDNA wiper Mix prior to reverse transcription. The cDNA was synthesized from 1 μg of total RNA using HiScript II qRT SuperMix II (Vazyme, R223). The reverse transcription reaction was performed under the following conditions: 50 °C for 15 min, and 85 °C for 5 s. RT-qPCR was performed in triplicate with 1 µL of diluted cDNA, 0.4 µL of primers, and 5 µL of 2 × ChamQ SYBR qPCR Master Mix (Vazyme, Q311) in a final volume of 10 µL made up with ddH_2_O. The use of GAPDH as a reference gene was justified by previous studies demonstrating its stable expression in multiple plant species [30,31,32]. Three independent biological replicates were analyzed for each treatment, with each biological replicate consisting of pooled tissue from three plants. Primer Premier 5.0 was used to design primers for the target genes (Supplementary Table S1). The specificity of all primers was confirmed by melting curve analysis, and amplification efficiencies were determined using standard curves. All reactions were performed in three biological replicates, and relative gene expression levels were calculated using the 2^−ΔΔCt^ method [33].

### 2.15. Statistical Analysis

All statistical analyses in this manuscript were derived from measurements of biologically independent samples. Statistical significance was determined using one-way analysis of variance (ANOVA), with Tukey’s HSD test for multiple group comparisons and Student’s *t*-test for two-group comparisons, as appropriate, and all analyses were performed using SPSS (version 25). A difference of *p* < 0.05 was considered significant. All figures were generated using GraphPad Prism (version 8.0.2).

## 3. Results

### 3.1. Sequence Properties and Predicted Characteristics of GbPHT Proteins

A total of 21 non-redundant phosphate transporter (GbPHT) candidate genes were initially identified from the *G. biloba* genome based on conserved domain and homology searches. These candidates were further evaluated based on their conserved domain architecture, phylogenetic relationships, protein integrity, and predicted transmembrane topology. These candidates were further evaluated based on their conserved domain architecture, phylogenetic relationships, protein integrity, and predicted transmembrane topology, and were subsequently classified into different PHT subfamilies and named according to their phylogenetic relationships with characterized PHT proteins and chromosomal positions. The physicochemical characteristics of the encoded proteins, including amino acid length, molecular weight, theoretical isoelectric point (pI), hydrophobicity, and predicted subcellular localization using CELLO, are summarized in Table 1.

The identified GbPHT proteins exhibited substantial variation in their physicochemical properties. The protein length ranged from 115 aa (GbPHT4-1) to 796 aa (GbPHO1-2), with predicted molecular weights ranging from 12.11 kDa to 91.49 kDa. The theoretical pI values varied from 5.94 to 9.91, indicating differences in protein charge characteristics among members of the family. The GRAVY index analysis showed that most GbPHT proteins exhibited positive hydrophobicity values, consistent with their predicted membrane-associated characteristics. Computational prediction by CELLO v.2.5 suggested a predominant plasma membrane localization for the GbPHT proteins. However, this prediction was not fully consistent with the experimentally characterized subcellular localization patterns of PHT proteins in other plant species. Therefore, the predicted localization should be interpreted with caution and requires experimental validation.

### 3.2. Chromosomal Localization and Phylogenetic Analysis of GbPHTs

Chromosomal localization analysis revealed that the 21 *GbPHT* genes were unevenly distributed across seven chromosomes of *G. biloba* (Figure 1a). Among them, chromosome 10 contained the largest number of *GbPHT* genes (nine members), whereas chromosome 2 contained only one *GbPHT* gene. Chromosomes 3, 4, 7, and 12 each harbored two *GbPHT* genes, while chromosome 9 contained three members. No *GbPHT* genes were identified on chromosomes 1, 5, 6, 8, or 11 (Figure 1a).

To investigate the evolutionary relationships of GbPHT proteins, a phylogenetic tree was constructed using 21 GbPHT proteins together with PHT proteins from *A. thaliana*, *O. sativa*, and *P. trichocarpa* (Figure 1b). The phylogenetic analysis classified PHT proteins into five conserved subfamilies: PHT1, PHT2, PHT3, PHT4, and PHO. Among the five subfamilies, the GbPHT4 subfamily represented the largest group, containing six members. The PHT1 and PHO subfamilies each contained five members, followed by *GbPHT3* with four members and *GbPHT2* with only one member. Comparative analysis showed that the number of members within each GbPHT subfamily was generally comparable with those identified in other plant species. For example, *GbPHT4* contained six members, similar to *A. thaliana* and *P. trichocarpa*, while *GbPHT2* consisted of a single member, consistent with the corresponding subfamilies in *A. thaliana*, *O. sativa*, and *P. trichocarpa*. Phylogenetic analysis further showed that several GbPHT proteins clustered more closely with their counterparts from *P. trichocarpa* than with those from *A. thaliana* and *O. sativa*, suggesting relatively high sequence similarity between the corresponding PHT proteins of *G. biloba* and *P. trichocarpa*.

### 3.3. Structural Characteristics and Conserved Domains of GbPHT Genes

To further characterize the structural diversity of the GbPHT family, conserved domains, gene structures, and conserved motifs were analyzed (Figure 2). Domain analysis using the NCBI Conserved Domain Database revealed that GbPHT proteins displayed distinct subfamily-specific domain organizations (Figure 2b). Members of the GbPHT1 and GbPHT4 subfamilies contained the Major Facilitator Superfamily (MFS) domain. In addition, GbPHT1 proteins possessed an SPX domain, forming a typical SPX-MFS domain structure. The GbPHT2 member contained a conserved PHO4 domain, whereas all GbPHT3 proteins harbored mitochondrial carrier family (MCF) domains. The PHO subfamily generally exhibited the characteristic SPX-EXS domain architecture; however, several members lacked the SPX domain, suggesting structural variation within this subfamily.

Gene structure analysis revealed substantial differences among GbPHT subfamilies (Figure 2c). Members within the GbPHT1 subfamily contained only one exon, whereas GbPHO and GbPHT4 subfamilies showed greater structural complexity. The exon number of GbPHO members ranged from two to sixteen, while GbPHT4 members contained one to thirteen exons. The GbPHT3 subfamily exhibited relatively conserved exon structures, containing three to six exons, while the GbPHT2 member contained four exons. Overall, exon–intron organization patterns were highly consistent with phylogenetic classification, with closely related members showing similar gene structures.

To further examine sequence conservation, conserved motifs were identified using MEME analysis. A total of 20 conserved motifs were detected among GbPHT proteins (Figure 2a). Distinct motif distribution patterns were observed among different subfamilies. Specifically, motifs 2, 3, 5, and 7−10 were mainly present in GbPHT1 proteins, while motif 17 was unique to GbPHT2, GbPHT3 members specifically contained motifs 18 and 19, whereas GbPHO proteins exhibited unique motif combinations including motifs 13 and 20. The GbPHT1 and GbPHT4 subfamilies shared several conserved motifs, including motifs 1 and 6. Overall, motif composition was highly conserved within each subfamily and corresponded well with phylogenetic relationships.

### 3.4. Cis-Regulator Elements in the Promoters of GbPHT Genes

To identify potential regulatory features of the *GbPHT* genes, the 2-kb upstream promoter regions of all *GbPHT* members were analyzed using the PlantCARE database. A total of 33 types of putative cis-regulatory elements were identified and classified into three major categories, including light-responsive elements, hormone-responsive elements, and stress-related elements (Figure 3). Among the identified hormone-responsive elements, several types associated with responses to phytohormones were detected, including abscisic acid-responsive elements (ABREs), methyl jasmonate-responsive elements (CGTCA motifs), auxin-responsive elements (AuxREs), salicylic acid-responsive elements (SAREs), and gibberellin-responsive elements (GAREs). Stress-related elements included low-temperature-responsive elements (LTRs), drought-responsive elements (DREs), and antioxidant-responsive elements (AREs). In addition, multiple light-responsive elements were identified in the promoter regions of *GbPHT* genes, suggesting that these genes may be potentially responsive to diverse environmental and hormonal cues.

### 3.5. Effects of Phosphorus Deficiency on the Growth and Physiological Characteristics of G. biloba Seedlings

To investigate the effects of phosphorus availability on the growth performance and physiological responses of *G. biloba*, seedlings grown under hydroponic conditions were subjected to normal phosphorus (200 μmol·L^−1^ KH_2_PO_4_, 200 P) and phosphorus-deficient conditions (0 μmol·L^−1^ KH_2_PO_4_, 0 P). After treatment, significant differences in growth phenotypes were observed between the two phosphorus regimes (Figure 4a). Phosphorus deficiency significantly inhibited root development in *Ginkgo* seedlings. Compared with seedlings grown under normal phosphorus conditions, phosphorus-deficient plants exhibited significantly reduced lateral root numbers and shorter primary roots (Figure 4b,c). In addition, phosphorus deficiency markedly suppressed aboveground growth, as indicated by significant reductions in leaf area, leaf fresh weight, and leaf dry weight (Figure 4d–f).

Phosphorus deficiency also significantly affected photosynthetic pigment accumulation. Compared with the control treatment, phosphorus-deficient seedlings showed significant decreases in chlorophyll a, chlorophyll b, carotenoid, and total chlorophyll contents (Figure 4g–j). These results demonstrate that phosphorus deficiency negatively affects both vegetative growth and photosynthetic pigment synthesis in *G. biloba* seedlings.

### 3.6. Effects of Phosphorus Deficiency on Photosynthetic Performance and Chlorophyll Fluorescence Characteristics in G. biloba

To further investigate the effects of phosphorus deficiency on photosynthetic performance in *G. biloba*, gas exchange parameters and chlorophyll fluorescence characteristics were systematically analyzed under normal phosphorus (200 P) and phosphorus deficiency (0 P) conditions. Compared with the normal phosphorus treatment, phosphorus deficiency significantly reduced the photosynthetic capacity of *G. biloba* leaves. The net photosynthetic rate (*Pn*), water use efficiency (WUE), and intercellular CO_2_ concentration (*Ci*) decreased by 69.90%, 77.17%, and 18.35%, respectively, under phosphorus-deficiency conditions (Figure 5c–e). In contrast, stomatal conductance (*Gs*) and transpiration rate (Tr) showed no significant differences between the two phosphorus treatments (Figure 5a,b). Chlorophyll fluorescence analysis further revealed that phosphorus deficiency adversely affected the photosynthetic apparatus. Fluorescence imaging showed obvious differences in leaf photosynthetic status between normal and phosphorus-deficient treatments (Figure 5g). In addition, the maximum photochemical efficiency of photosystem II (*Fv/Fm*) was significantly reduced under phosphorus deficiency compared with the control treatment (Figure 5f). These results demonstrate that phosphorus deficiency substantially impairs photosynthetic efficiency and PSII photochemical performance in *G. biloba* seedlings.

### 3.7. Effects of Phosphorus Deficiency on Medicinal Metabolite Accumulation

Flavonoids and terpene lactones are major medicinal compounds determining the pharmaceutical value of *G. biloba*. To investigate the effects of phosphorus availability on secondary metabolite accumulation, flavonoid and terpene lactone contents were measured in leaves and roots under normal and phosphorus-deficient conditions. Phosphorus deficiency treatment significantly increased flavonoid content in both leaves and roots. The total flavonoid content in leaves reached 13.72 mg g^−1^ under phosphorus deficiency, representing a 1.46-fold increase compared with the normal phosphorus treatment. Similarly, root flavonoid content increased to 6.04 mg g^−1^, which was 1.58-fold higher than that in the control group (Figure 6a). Phosphorus deficiency also significantly increased terpene lactone contents in roots. Compared with normal phosphorus conditions, the contents of ginkgolide A, ginkgolide B, ginkgolide C, and bilobalide increased by 64.28%, 89.21%, 100%, and 56.6%, respectively, resulting in a 66.68% increase in total terpene lactone content. However, no significant differences were observed in terpene lactone contents in leaves between the two phosphorus treatments (Figure 6b–f). In addition to changes in secondary metabolites, phosphorus deficiency significantly increased hydrogen peroxide (H_2_O_2_) content and superoxide dismutase (SOD) activity in roots (Figure 6g,h), suggesting that phosphorus-deficiency treatment altered redox homeostasis in *G. biloba* seedlings.

### 3.8. Effects of Phosphorus Deficiency on the Expression of Structural Genes Involved in Flavonoid and Terpene Lactone Biosynthesis

Consistent with the increased flavonoid accumulation observed under phosphorus-deficiency conditions, several structural genes associated with flavonoid biosynthesis were significantly upregulated after phosphorus-deficiency treatment. Specifically, the expression levels of chalcone synthase *(GbCHS*; *Gb_19002* and *Gb_20355*), flavonol synthase *(GbFLS*; *Gb_14031*), and flavonoid 3′-hydroxylase *(GbF3′H*; *Gb_19792*) were significantly increased compared with those under normal phosphorus conditions (Figure 7a–e). Similarly, genes involved in terpene lactone biosynthesis were also responsive to phosphorus deficiency. The expression levels of key upstream pathway genes, including acetyl-CoA C-acetyltransferase (*GbAACT1*; *Gb_28733*), *GbAACT2* (*Gb_04657*), *GbAACT3* (*Gb_04658*), and 1-Deoxy-D-Xylulose-5-Phosphate Synthase (*GbDXS1*; *Gb_34070*), were significantly induced under phosphorus-deficiency treatment (Figure 7f–j). These results indicate that phosphorus deficiency altered the expression of several structural genes associated with flavonoid and terpene lactone biosynthetic pathways, which may contribute to the increased accumulation of these metabolites under phosphorus deficiency conditions.

### 3.9. Expression Profiles of GbPHT Genes in Response to Phosphorus Deficiency

To investigate the transcriptional responses of *GbPHT* genes to phosphorus deficiency, the expression patterns of selected *GbPHT* members were analyzed in leaves and roots of *G. biloba* seedlings after 34 days of phosphorus-deficiency treatment using qRT-PCR (Figure 8). The expression profiles of *GbPHT* genes exhibited distinct tissue-specific responses to phosphorus deficiency. In roots, most detected *GbPHT* genes showed increased expression levels under phosphorus deficiency. However, the transcript levels of *GbPHT4-3* (*Gb_13175*), *GbPHT4-4* (*Gb_22574*), and *GbPHO1-1* (*Gb_34493*) were significantly reduced, whereas *GbPHT4-6* (*Gb_18639*) and *GbPHO1-4* (*Gb_22129*) showed no significant changes compared with the control treatment. In leaves, only a limited number of *GbPHT* genes exhibited transcriptional induction under phosphorus deficiency. Among them, *GbPHT1-1* (*Gb_05489*) and *GbPHT1-2* (*Gb_05490*) showed significant upregulation after phosphorus-deficiency treatment. In contrast, the expression levels of *GbPHT1-5* (*Gb_05492*), *GbPHT3-3* (*Gb_04962*), and *GbPHO1-4* (Gb_07356) remained unchanged, while several other *GbPHT* members showed reduced expression levels under phosphorus deficiency. Overall, *GbPHT* genes displayed diverse and tissue-dependent transcriptional responses to phosphorus deficiency, with *GbPHT1* members showing stronger induction patterns, particularly in leaves, suggesting their potential involvement in the response to phosphorus deficiency.

### 3.10. Functional Characterization of GbPHT1-1 in Regulating Flavonoid Accumulation in G. biloba Leaves

To investigate the potential role of *GbPHT1-1* in flavonoid accumulation, an *Agrobacterium*-mediated transient overexpression system was employed in intact *G. biloba* leaves. Five days after transformation, qRT-PCR analysis confirmed that the transcript abundance of GbPHT1-1 was significantly increased by approximately 2- to 4-fold in *GbPHT1-1*-overexpressing leaves compared with the empty vector control (Figure 9a), indicating successful transient overexpression. Further analysis showed that transient overexpression of *GbPHT1-1* significantly enhanced flavonoid accumulation, with total flavonoid content increasing by 17.76% compared with the control (Figure 9b). In addition, the expression levels of key flavonoid biosynthetic structural genes, including *GbCHS*, *GbFLS*, and *GbF3′H*, were significantly elevated in *GbPHT1-1*-overexpressing leaves (Figure 9c–f). These results demonstrate that *GbPHT1-1* positively affects flavonoid accumulation in *G. biloba* leaves and is associated with enhanced expression of flavonoid biosynthetic genes.

## 4. Discussion

### 4.1. Evolutionary Characteristics of the Ginkgo PHT Family and Their Adaptation to Phosphorus Deficiency

Phosphate transporters (PHTs) are essential components of the phosphate acquisition and distribution system, and their evolutionary diversification is closely associated with plant adaptation to heterogeneous phosphorus availability [3]. In the present study, 21 *GbPHT* genes were identified from the *G. biloba* genome and classified into five conserved subfamilies, including PHT1, PHT2, PHT3, PHT4, and PHO, with five, one, four, six and five members in each subfamily, respectively (Figure 1). The presence of all major PHT subfamilies in *Ginkgo* suggests that the major classes of phosphate transporters have been retained during seed plant evolution.

Although *G. biloba* possesses a large and complex genome containing 12 chromosome pairs and an estimated genome size of approximately 10.2 Gb [34], the number of *GbPHT* genes is relatively lower than that reported in several angiosperm species, such as *A. thaliana* (23 members) and rice (24 members) [11,35,36]. This observation is consistent with previously reported whole-genome characteristics indicating that *Ginkgo* genome gigantism was predominantly driven by the extensive accumulation of repetitive non-coding elements (such as LTR retrotransposons) rather than recent whole-genome duplications [34]. Thus, the relatively limited expansion of the *GbPHT* family compared with genome size may reflect a comparatively conserved phosphate transport repertoire in *Ginkgo*. As a long-lived woody species that has survived under diverse and often nutrient-limited environments, *G. biloba* may have developed regulatory mechanisms that enable efficient phosphorus homeostasis through the functional coordination of existing PHT members rather than extensive gene family expansion. This possibility may represent an adaptive feature of phosphorus.

Previous studies have shown that the PHT1 subfamily generally represents the largest group among plant phosphate transporters, as PHT1 members play predominant roles in high-affinity phosphate uptake from the soil and phosphorus deficiency responses. For example, *Arabidopsis* contains nine *AtPHT1* members, while rice contains 13 *OsPHT1* genes [35,36,37]. Interestingly, unlike these angiosperm models where PHT1 predominates numerically, the *GbPHT4* subfamily constitutes the largest subgroup within the identified *GbPHT* family in *Ginkgo*, containing six members that are mainly distributed on chromosome 10 (Figure 1). This atypical expansion suggests that tandem duplications may have driven GbPHT4 diversification in *Ginkgo*. Given that PHT4 proteins primarily mediate organellar Pi transport, this divergence implies that *G. biloba* may rely more heavily on intracellular Pi remobilization and organellar metabolic homeostasis than on expanding root uptake capacity under phosphorus limitation.

Promoter cis-element analysis further revealed that *GbPHT* genes contain abundant light-, stress-, and phytohormone-responsive elements, including methyl jasmonate (MeJA), abscisic acid (ABA), and salicylic acid (SA)-responsive motifs (Figure 3). These findings suggest that *GbPHT* transcription is regulated not only by phosphorus availability but also by complex interactions between nutrient signaling, environmental cues, and hormonal pathways. Previous studies in sweet potato showed that salt stress reduced phosphorus uptake capacity and suppressed the expression of PHT genes [38], indicating that phosphate transport systems may integrate multiple environmental stress signals. Therefore, PHT proteins may represent downstream regulatory components that rapidly adjust phosphate acquisition and distribution according to changing environmental conditions.

Additionally, computational prediction suggested a predominant plasma membrane localization for the GbPHT proteins analyzed in this study. However, evidence from well-characterized plant species indicates that different PHT subfamilies exhibit distinct subcellular localization patterns (Table 1). For example, PHT2, PHT3, and PHT4 proteins are predominantly associated with chloroplasts, mitochondria, and Golgi/plastid compartments, respectively [3]. This discrepancy may reflect limitations of sequence-based prediction, particularly for divergent gymnosperm proteins and truncated sequences. Therefore, the CELLO predictions should be considered preliminary, and experimental validation using GFP-tagged proteins will be required to confirm the precise subcellular localization of GbPHT proteins. In addition, GbPHT4-1 (115 aa), GbPHO1-3 (139 aa), and GbPHO1-4 (134 aa) are unusually short compared with typical members of their respective families. InterProScan analysis revealed that GbPHT4-1 retains an MFS-related domain across nearly its entire sequence, whereas GbPHO1-3 and GbPHO1-4 contain only partial EXS domains, suggesting that these sequences may differ in structural completeness. Their atypical features may reflect compact proteins, incomplete gene models, or potentially non-functional variants, and further experimental validation is needed to clarify their biological significance.

### 4.2. Low Phosphorus Suppresses Growth Through Photosynthetic Limitation

Phosphorus deficiency is one of the major nutritional constraints limiting plant growth because phosphate participates directly in energy metabolism, nucleic acid synthesis, membrane formation, and carbon assimilation [3]. In many herbaceous crops and angiosperms, phosphorus deficiency triggers compensatory root foraging strategies, including enhanced primary root elongation, increased lateral root formation, and higher root hair density to improve soil phosphate acquisition [39,40]. However, our results showed that severe phosphorus deficiency significantly inhibited both root and shoot development in *G. biloba*, as evidenced by reductions in primary root length, lateral root number, leaf area, fresh weight, and dry weight (Figure 4).

The distinct response pattern observed in *Ginkgo* under phosphorus deficiency might be linked to resource allocation strategies often found in perennial woody plants. Unlike rapidly growing annual crops that frequently enhance root exploration under nutrient limitation, woody plants often exhibit more conservative resource allocation strategies. Under severe phosphorus deficiency, insufficient Pi availability is documented to impair ATP production, phosphorylated metabolite synthesis, and carbon metabolism [9], which likely constrained vegetative growth in our study. Consequently, rather than actively investing in root system expansion, *Ginkgo* seedlings may theoretically adjust their physiology toward basal metabolic maintenance under phosphorus-deficiency conditions, although the exact energetic and carbon partitioning mechanisms require direct physiological and biochemical verification.

Photosynthesis is particularly sensitive to phosphorus availability because inorganic phosphate is directly involved in photophosphorylation, ATP synthesis, triose phosphate utilization, and regeneration of ribulose-1,5-bisphosphate (RuBP) in the Calvin-Benson cycle [41]. In the present study, phosphorus deficiency significantly reduced net photosynthetic rate (*Pn*), water use efficiency (WUE), and intercellular CO_2_ concentration (*Ci*). Interestingly, stomatal conductance (*Gs*) and transpiration rate (*Tr*) remained relatively stable (Figure 5), suggesting that photosynthetic inhibition may have been primarily associated with non-stomatal limitations rather than stomatal closure.

The reduction in photosynthetic performance was further supported by significant decreases in chlorophyll a, chlorophyll b, carotenoid contents, and maximum PSII photochemical efficiency (*Fv/Fm*). Phosphorus deficiency can disrupt chloroplast structure and membrane lipid metabolism, resulting in impaired photosynthetic electron transport and altered reactive oxygen species (ROS) production (Figure 5). Therefore, the reduced photosynthetic capacity observed in *Ginkgo* under phosphorus deficiency likely represents a consequence of impaired chloroplast metabolism and energy limitation rather than a simple reduction in carbon dioxide diffusion.

### 4.3. Phosphorus Deficiency Promotes Medicinal Metabolite Accumulation

Phosphorus deficiency did not simply cause metabolic suppression but was associated with increased accumulation of important medicinal metabolites in *G. biloba*. Although phosphorus-deficiency treatment significantly inhibited growth and photosynthetic capacity, flavonoid contents increased by 1.46-fold and 1.58-fold in leaves and roots, respectively (Figure 6). Consistently, the expression levels of several structural genes associated with flavonoid biosynthetic pathways, including *GbCHS*, *GbFLS* and *GbF3′H*, were significantly induced under phosphorus deficiency (Figure 7). Similar increases in flavonoid accumulation under phosphorus deficiency have been reported in several plant species, including white lupin, *Arabidopsis*, maize, *Malus*, and *Torreya grandis* [42,43,44,45,46], suggesting that increased flavonoid accumulation may represent a common metabolic response to nutrient limitation. The accumulation of flavonoids under phosphorus deficiency may provide multiple adaptive advantages. Flavonoids can function as antioxidants to eliminate excessive ROS generated under stress conditions and protect cellular components from oxidative damage [47]. In addition, root-secreted flavonoids can influence rhizosphere microbial communities by promoting beneficial plant-microbe interactions, potentially improving phosphorus acquisition efficiency [48]. Therefore, increased flavonoid accumulation under phosphorus deficiency may represent an adaptive response associated with stress tolerance and nutrient acquisition. In addition to flavonoids, phosphorus availability also significantly influenced terpene lactone metabolism in *Ginkgo*. Terpene lactones, including ginkgolides and bilobalide, are major bioactive compounds responsible for the medicinal properties of *Ginkgo* extracts [19]. In the present study, phosphorus deficiency markedly increased total terpene lactone content in roots by 66.68%, accompanied by significant increases in ginkgolide A, B, C, and bilobalide contents (Figure 6). Meanwhile, genes involved in the early steps of terpene biosynthesis pathways, including *GbAACT1/2/3* and *GbDXS1*, were upregulated under phosphorus deficiency (Figure 7), suggesting that phosphorus deficiency may enhance the transcriptional activity of the upstream terpene biosynthetic pathway and contribute to the accumulation of terpenoid metabolites.

### 4.4. GbPHT1-1 Represents a Potential Link Between Phosphate Transport and Flavonoid Biosynthesis

The PHT1 family encodes high-affinity phosphate transporters essential for root phosphorus uptake and intercellular phosphorus transport under phosphorus deficiency. Members of this family, such as *AtPHT1;1* in *Arabidopsis* [11], are typically upregulated under phosphorus deficiency to enhance phosphorus acquisition. In our study, both *GbPHT1-1* was significantly induced under phosphorus deficiency in roots and leaves (Figure 8), suggesting their involvement in the phosphorus deficiency response of *G. biloba*. We selected *GbPHT1-1* for further functional characterization. Transient overexpression of *GbPHT1-1* in intact *Ginkgo* leaves provided evidence that this transporter may have functions beyond phosphate acquisition. Overexpression of *GbPHT1-1* significantly increased total flavonoid content by 17.76% and enhanced the expression of flavonoid biosynthetic genes, including *GbCHS*, *GbFLS*, and *GbF3′H* (Figure 9). These results support a potential association between *GbPHT1-1* and flavonoid accumulation during phosphorus deficiency.

Although PHT1 proteins have traditionally been considered phosphate transporters, increasing evidence indicates that phosphate transport systems are integrated into broader nutrient signaling networks [3]. PHT1 activity can influence intracellular phosphate status, which subsequently affects phosphate deficiency signaling pathways involving PHR1, SPX proteins, and downstream transcriptional regulators [49,50]. Therefore, the effect of *GbPHT1-1* on flavonoid accumulation may not result from direct regulation of flavonoid biosynthetic genes, but rather from altered phosphate homeostasis and activation of downstream metabolic adaptation pathways. The promoter region of *GbPHT1-1* contains multiple hormone-responsive cis-elements, including MeJA-responsive motifs (Figure 3), suggesting that jasmonate signaling may be involved in the regulation of *GbPHT1-1*. Given the close association between jasmonate signaling and stress-induced secondary metabolism, we hypothesize that *GbPHT1-1* may mediate phosphate homeostasis and interact with jasmonate signaling to regulate flavonoid and other medicinal metabolite biosynthesis. This hypothesis, including the potential involvement of PHR1/SPX-mediated phosphate signaling and jasmonate-responsive transcription factors, remains to be experimentally validated in future studies.

## 5. Conclusions

In summary, this study identified and characterized the phosphate transporter (PHT) family in *G. biloba* and revealed its potential involvement in the response to phosphate deficiency. The *G. biloba* PHT family comprises 21 members categorized into five subfamilies: PHT1, PHT2, PHT3, PHT4, and PHO. Promoter analysis revealed an abundance of light-, hormone-, and stress-responsive elements, indicating that GbPHT genes may respond to diverse environmental and hormonal signals. Phosphate deficiency significantly inhibited seedling growth and photosynthetic performance but promoted flavonoid accumulation in both leaves and roots and enhanced root-specific terpene trilactone biosynthesis. Furthermore, transient overexpression of *GbPHT1-1* enhanced flavonoid accumulation and upregulated the expression of key structural genes, suggesting that *GbPHT1-1* may serve as a functional bridge linking phosphate signaling to medicinal secondary metabolism. Future studies should combine transcriptomics, metabolomics, and genetic approaches to elucidate the regulatory mechanisms linking phosphate deficiency signaling with secondary metabolism and to clarify the specific functions of individual PHT members in phosphate acquisition and utilization and bioactive compound biosynthesis. Such knowledge may provide a theoretical basis for optimizing phosphorus management and improving the quality of *G. biloba* medicinal resources.

## Figures and Tables

**Figure 1:**
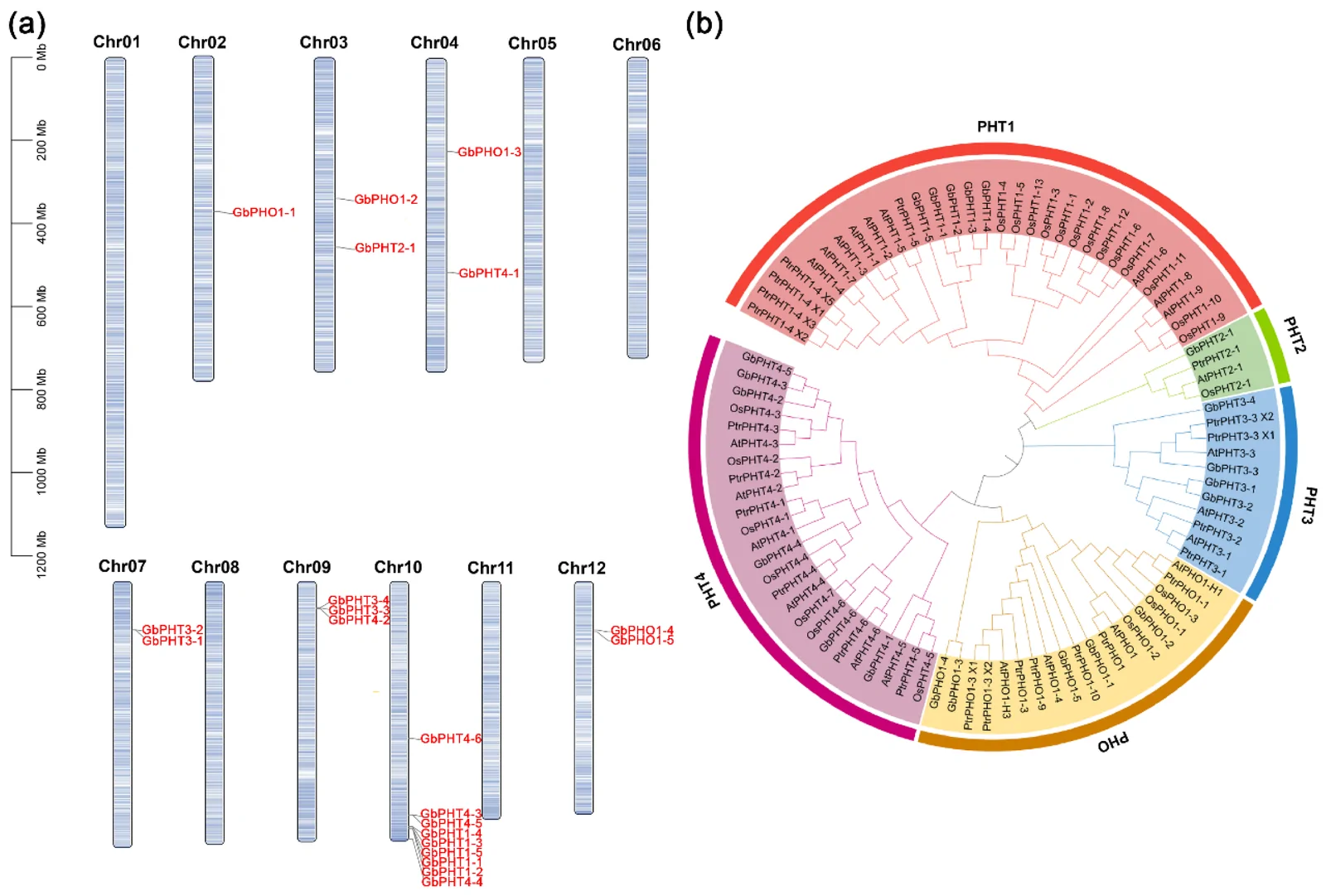
Chromosomal locations and an unrooted phylogenetic tree of *GbPHT* family members. (**a**) Distribution of *GbPHT* genes on *G. biloba* chromosomes. The y-axis represents chromosome length, with Chr1-Chr12 representing the twelve *G. biloba* chromosomes. The red text denotes the 21 *GbPHT* genes in *G. biloba*. (**b**) The protein sequences of PHTs from *G. biloba*, *A. thaliana*, *O. sativa* and *P. trichocarpa* were used to generate an unrooted neighbour-joining phylogenetic tree with a bootstrap set at 1000 replicates. Different colors correspond to each subfamily within the PHT family.

**Figure 2:**
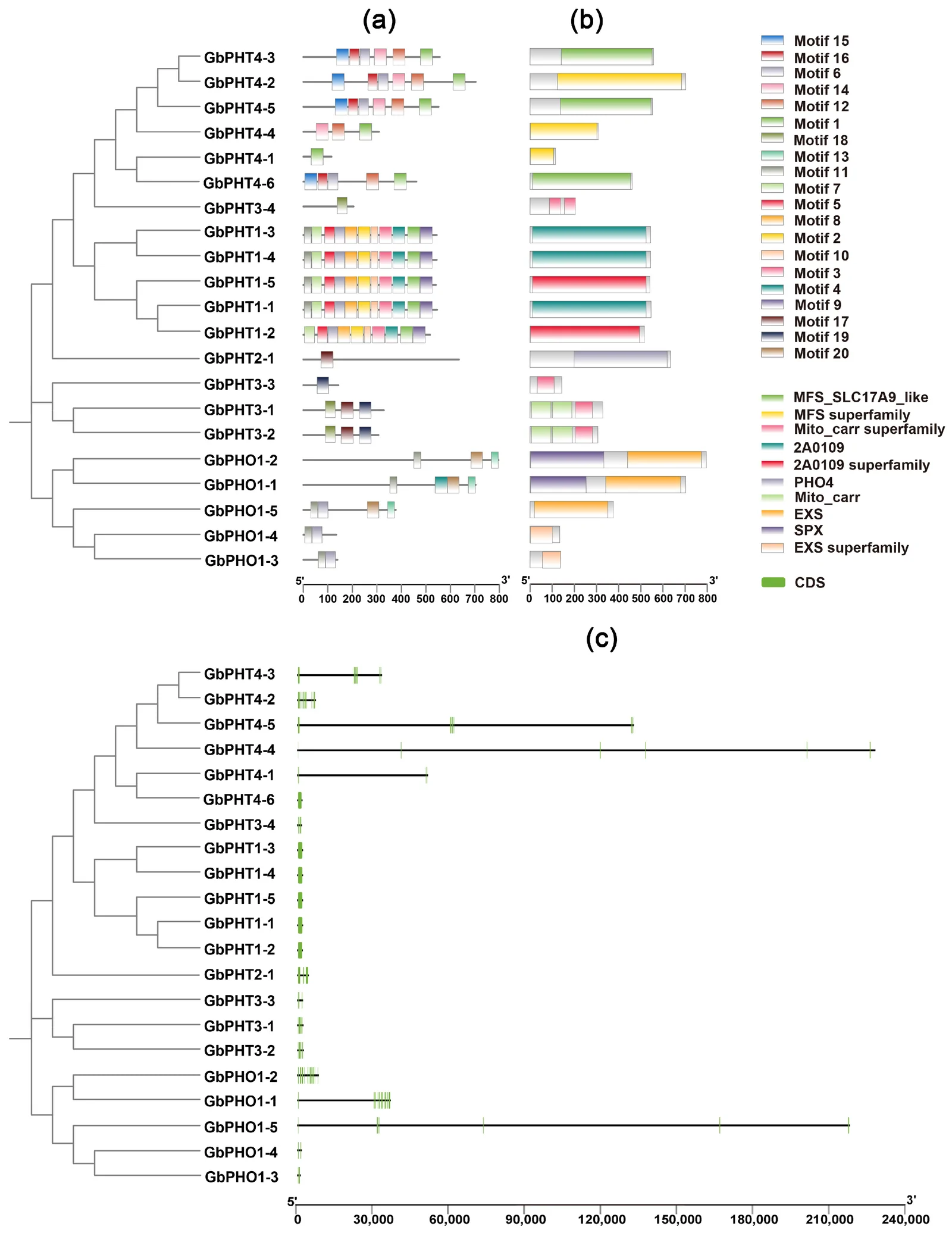
Gene structure of the GbPHT family in *G. biloba*. (**a**) The MEME server was used to predict conserved motifs in PHT proteins; a total of 20 conserved motifs were detected. The scale at the bottom of each figure represents the length of the protein and nucleotide sequences of each PHT family member; (**b**) The number of conserved domains in each subfamily was analyzed via the NCBI CDD by submitting corresponding protein sequences; (**c**) The distribution and number of exons and introns in each PHT family member.

**Figure 3:**
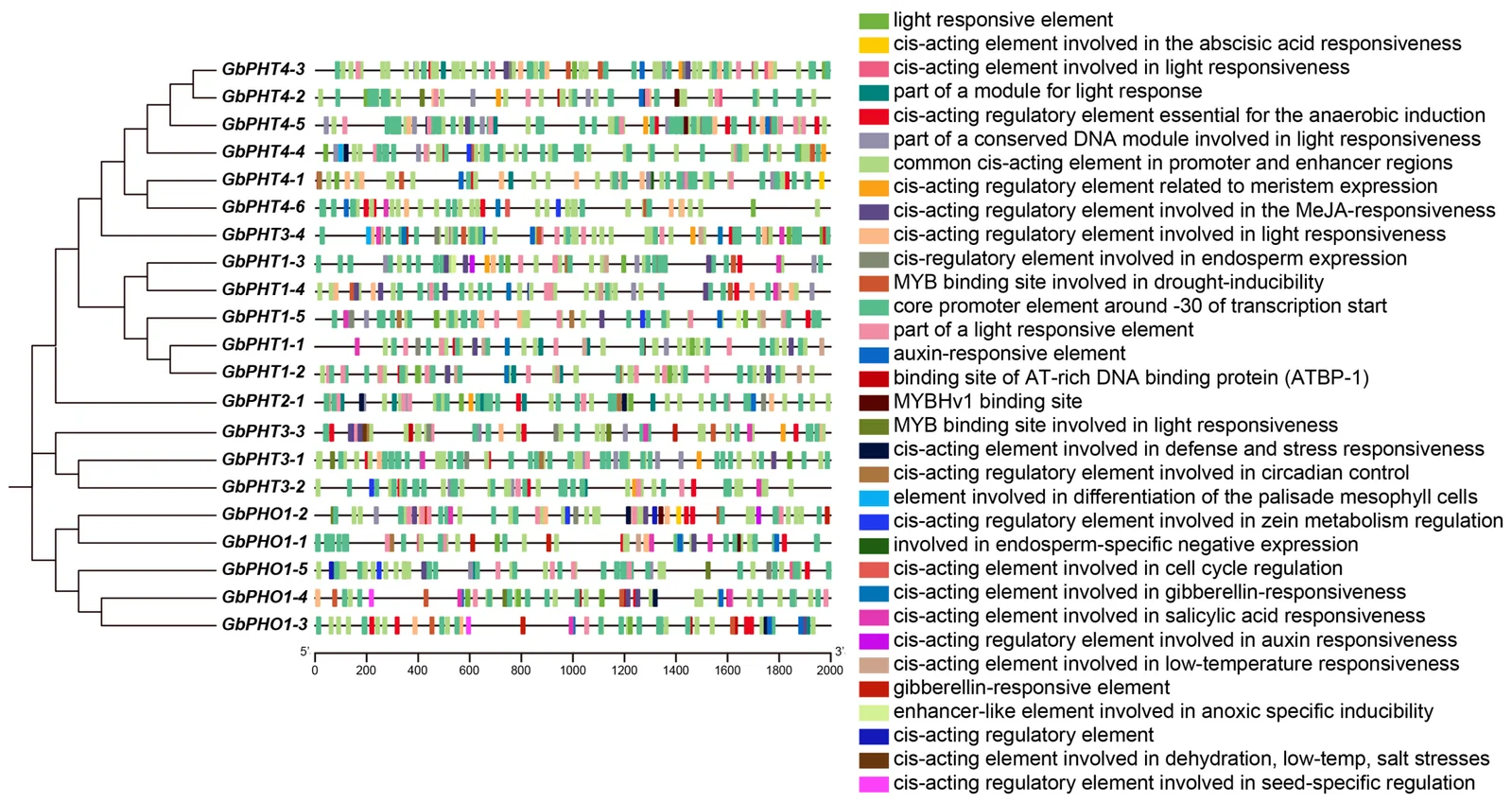
Cis-acting elements in the promoters of *GbPHT* family members.

**Figure 4:**
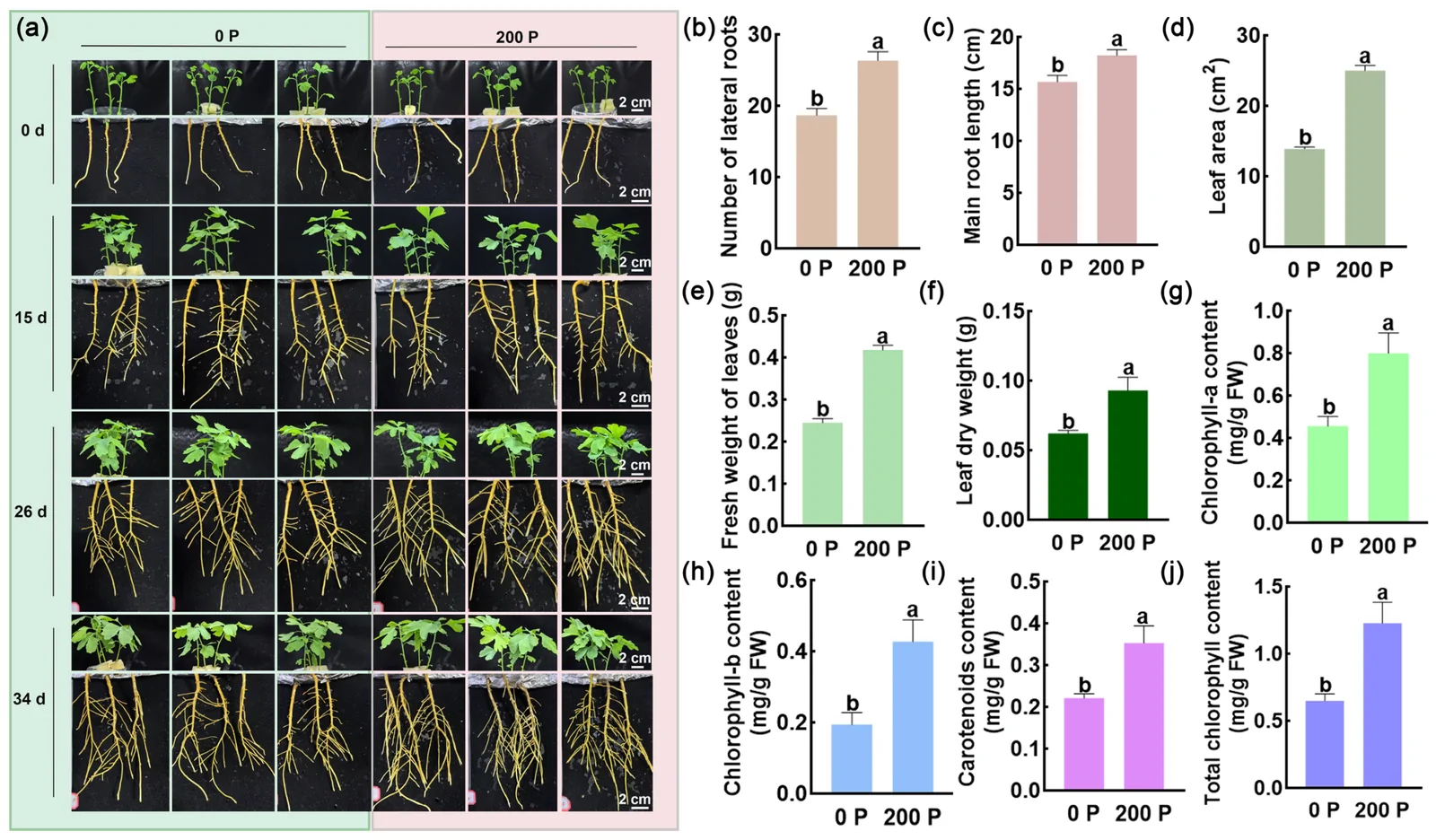
Phenotypic and physiological responses of *G. biloba* seedlings under different phosphorus conditions. (**a**) Morphological phenotypes of the shoots and roots of *G. biloba* seedlings grown under normal phosphorus (200 P) and phosphorus deficiency (0 P) conditions; (**b**) Lateral root number; (**c**) Primary root length; (**d**) Leaf area; (**e**) Leaf fresh weight; (**f**) Leaf dry weight; (**g**) Chlorophyll *a* content; (**h**) Chlorophyll *b* content; (**i**) Carotenoid content; (**j**) Total chlorophyll content. Data are presented as means ± SD of three biological replicates (*n* = 3). Different letters indicate significant differences at *p* < 0.05.

**Figure 5:**
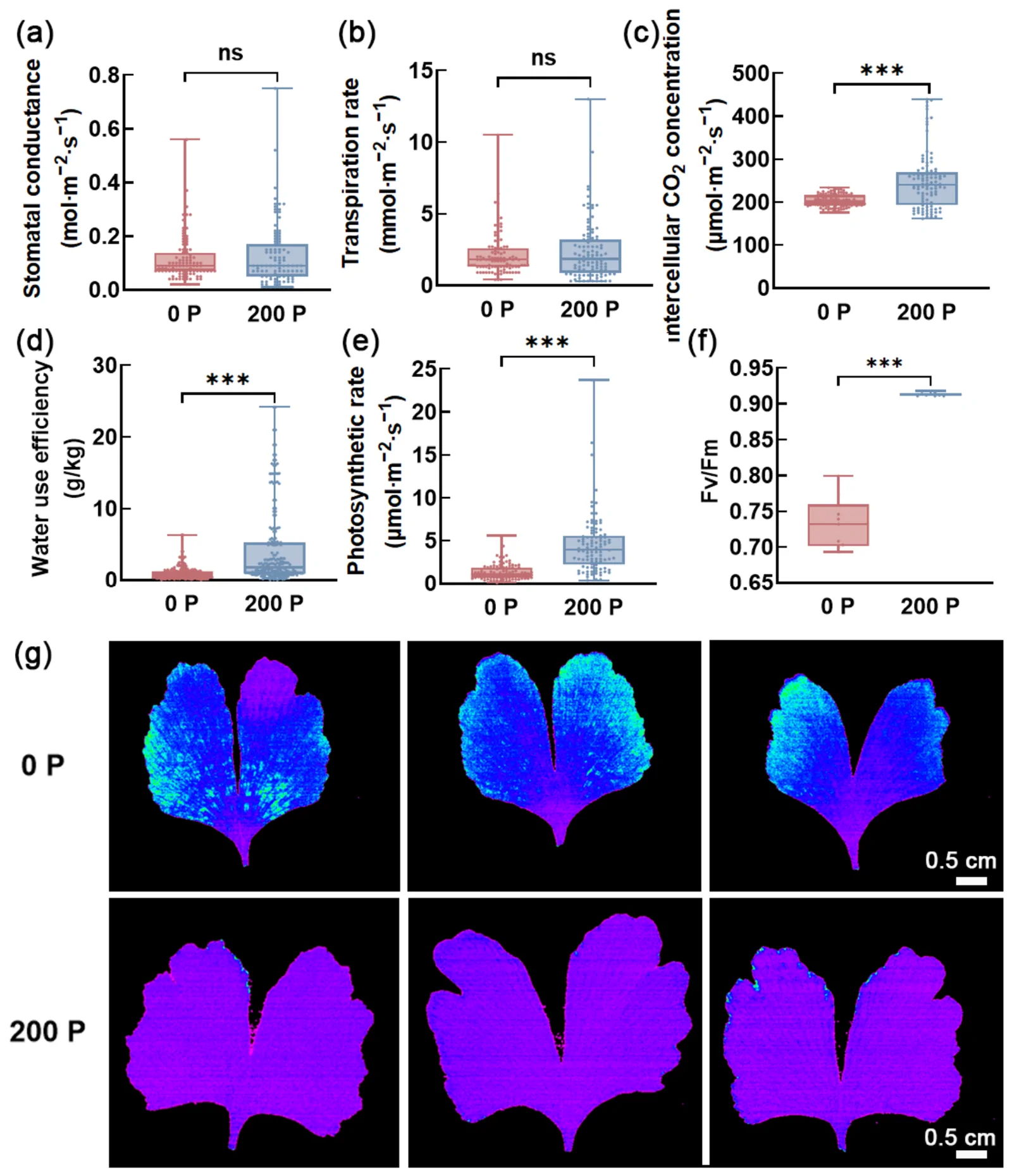
Effects of phosphorus deficiency on photosynthetic parameters and chlorophyll fluorescence characteristics in *G. biloba*. (**a**) Stomatal conductance (*Gs*); (**b**) Transpiration rate (*Tr*); (**c**) Intercellular CO_2_ concentration (Ci); (**d**) Water use efficiency (WUE); (**e**) Net photosynthetic rate (*Pn*); (**f**) Maximum photochemical efficiency of PSII (*Fv/Fm*); (**g**) Chlorophyll fluorescence imaging under 200 P and 0 P treatment. For each treatment, three representative images corresponding to three independent biological replicates are show. Asterisks (***) denote statistical significance at *p* < 0.001; ns denotes not significant.

**Figure 6:**
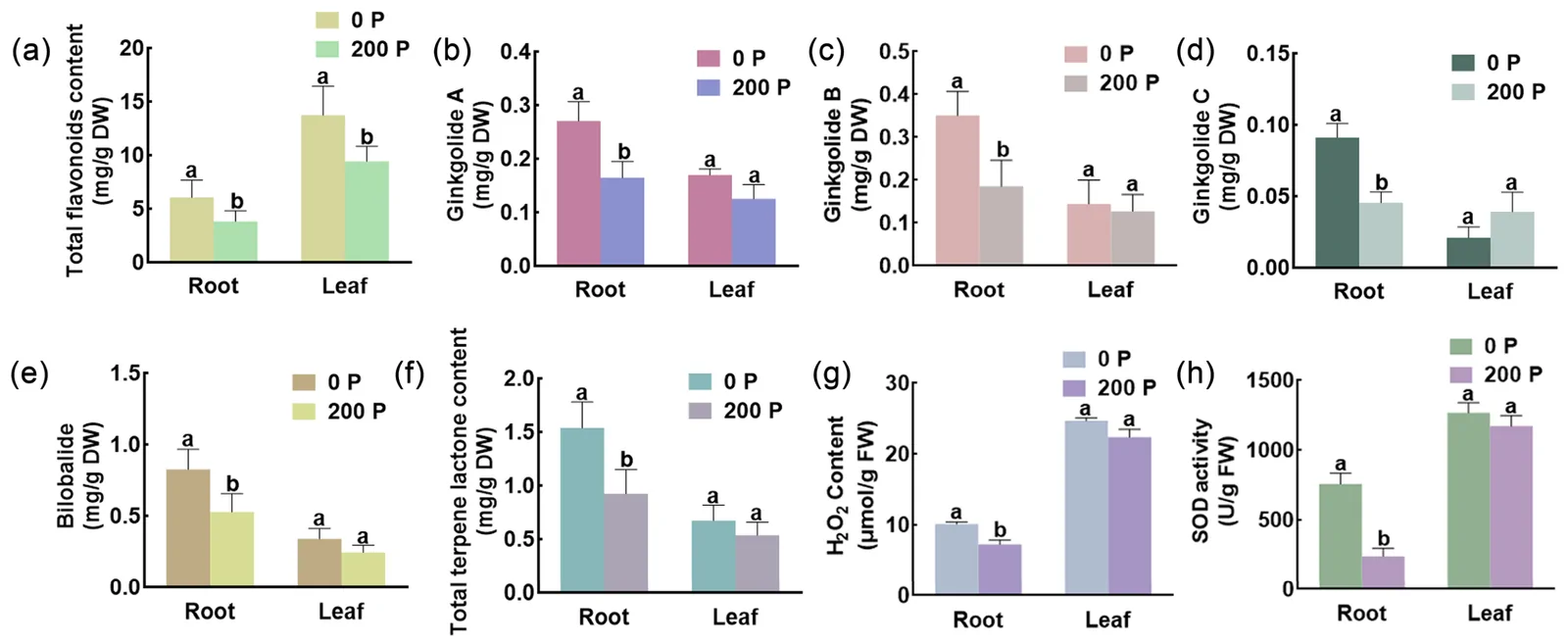
Effects of phosphorus deficiency on medicinal metabolite accumulation in *G. biloba*. (**a**) Total flavonoid content in leaves and roots; (**b**) ginkgolide A content; (**c**) ginkgolide B content; (**d**) ginkgolide C content; (**e**) bilobalide content; (**f**) total terpene lactone content; (**g**) H_2_O_2_ content of roots; (**h**) SOD activity of roots. Data are presented as the mean ± standard deviation (SD) of three independent biological replicates (*n* = 3). Different letters presented above the bars indicate the significant differences (One-way ANOVA statistical analysis, *p* < 0.05).

**Figure 7:**
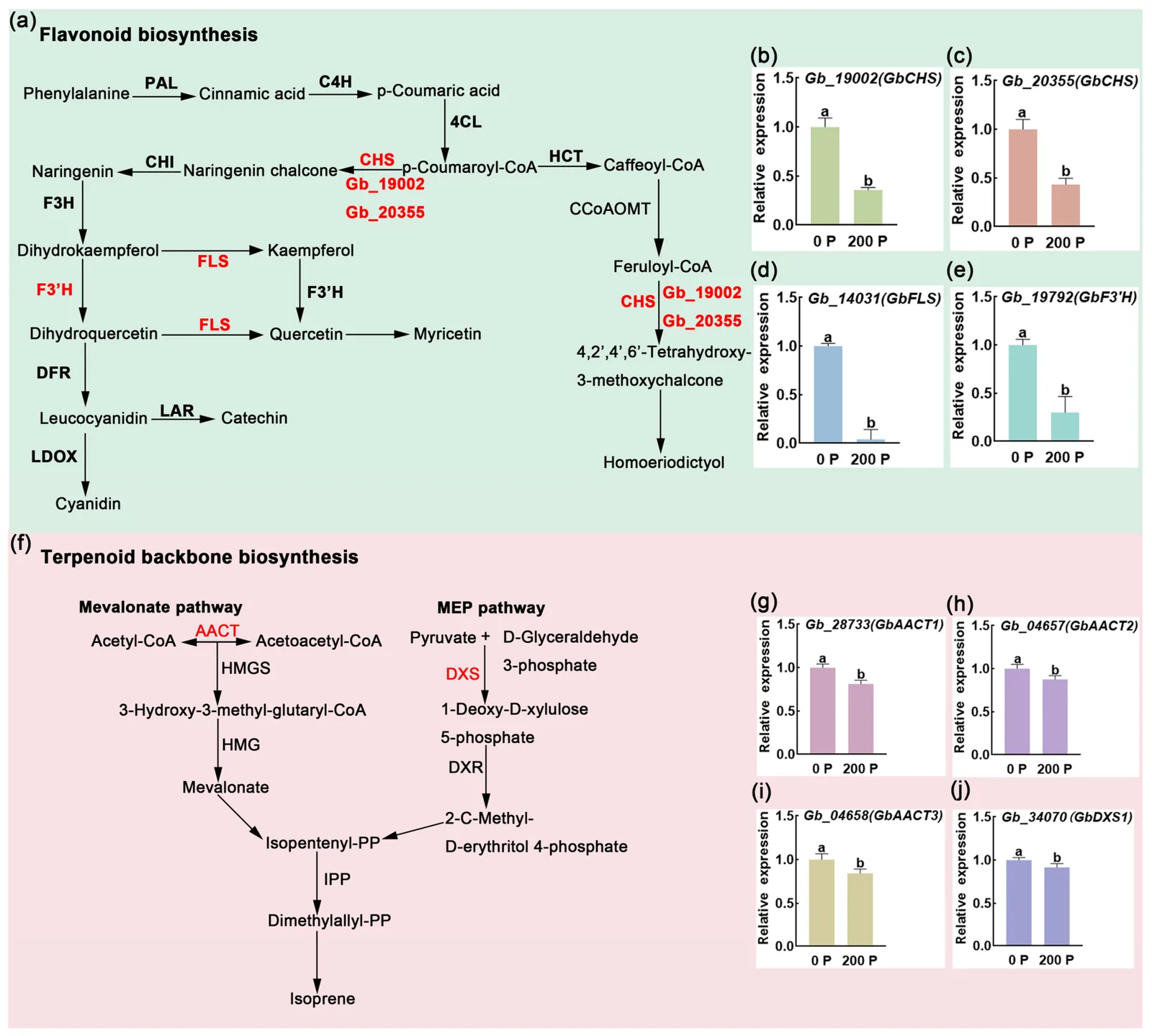
Expression analysis of structural genes involved in flavonoid and terpene lactone biosynthesis under phosphorus deficiency. (**a**) Schematic representation of the flavonoid biosynthetic pathway; (**b**–**e**) Expression levels of flavonoid biosynthetic genes under different P conditions; (**f**) Schematic representation of the terpene lactone biosynthetic pathway; (**g**–**j**) Expression levels of terpene lactone biosynthetic genes under different P conditions. The genes highlighted in red were selected for quantitative analysis. Data are presented as the mean ± standard deviation (SD) of three independent biological replicates (*n* = 3). Different letters presented above the bars indicate the significant differences (One-way ANOVA statistical analysis, *p* < 0.05).

**Figure 8:**
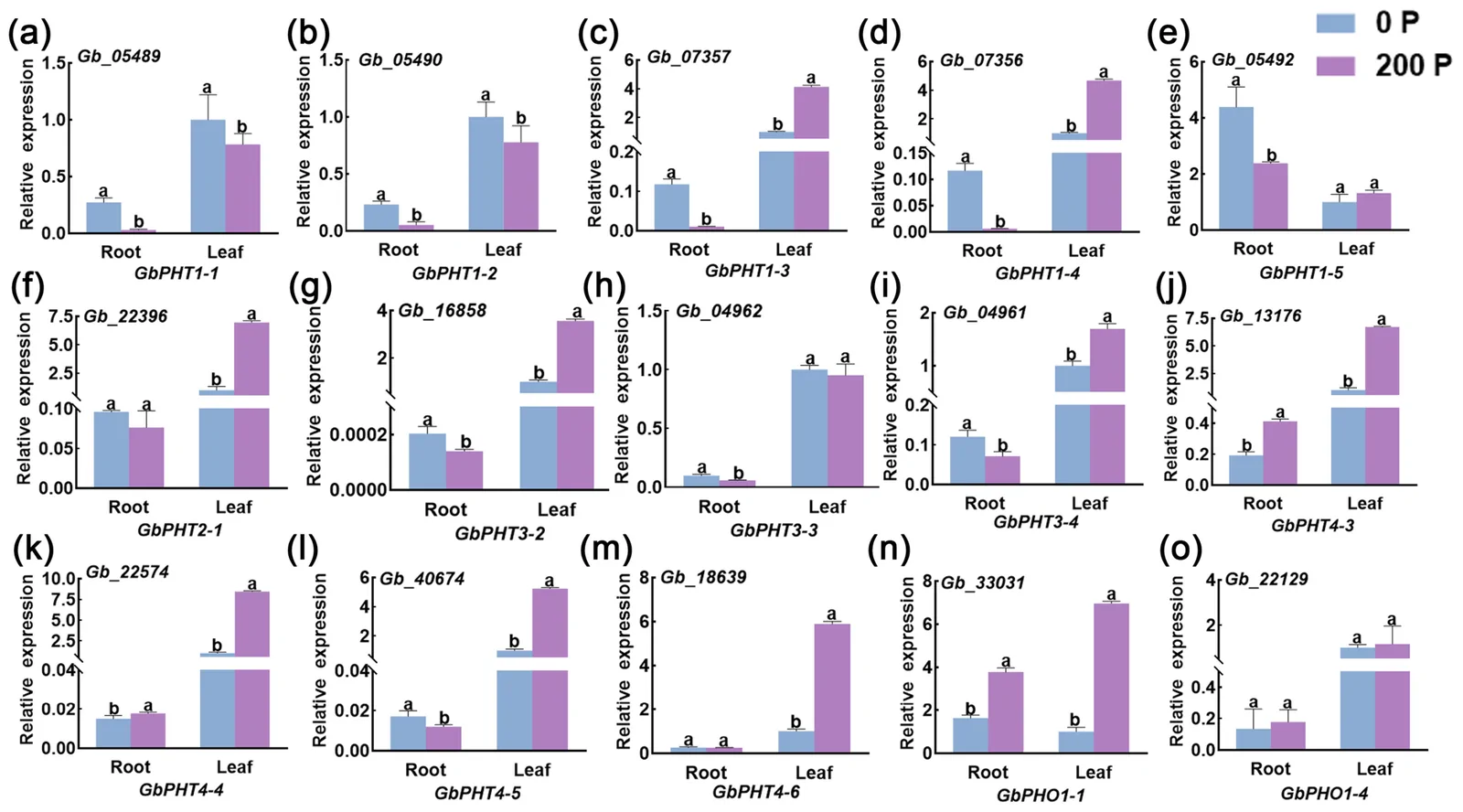
Expression patterns of *GbPHT* genes under phosphorus deficiency. (**a**–**o**) Relative expression levels of *GbPHT* genes under varying phosphorus conditions determined by qRT-PCR. In the bar graphs, blue bars correspond to the 0 P condition, and purple bars correspond to the 200 P condition. Data are presented as the mean ± standard deviation (SD) of three independent biological replicates (*n* = 3). Different letters presented above the bars indicate the significant differences (One-way ANOVA statistical analysis, *p* < 0.05).

**Figure 9:**
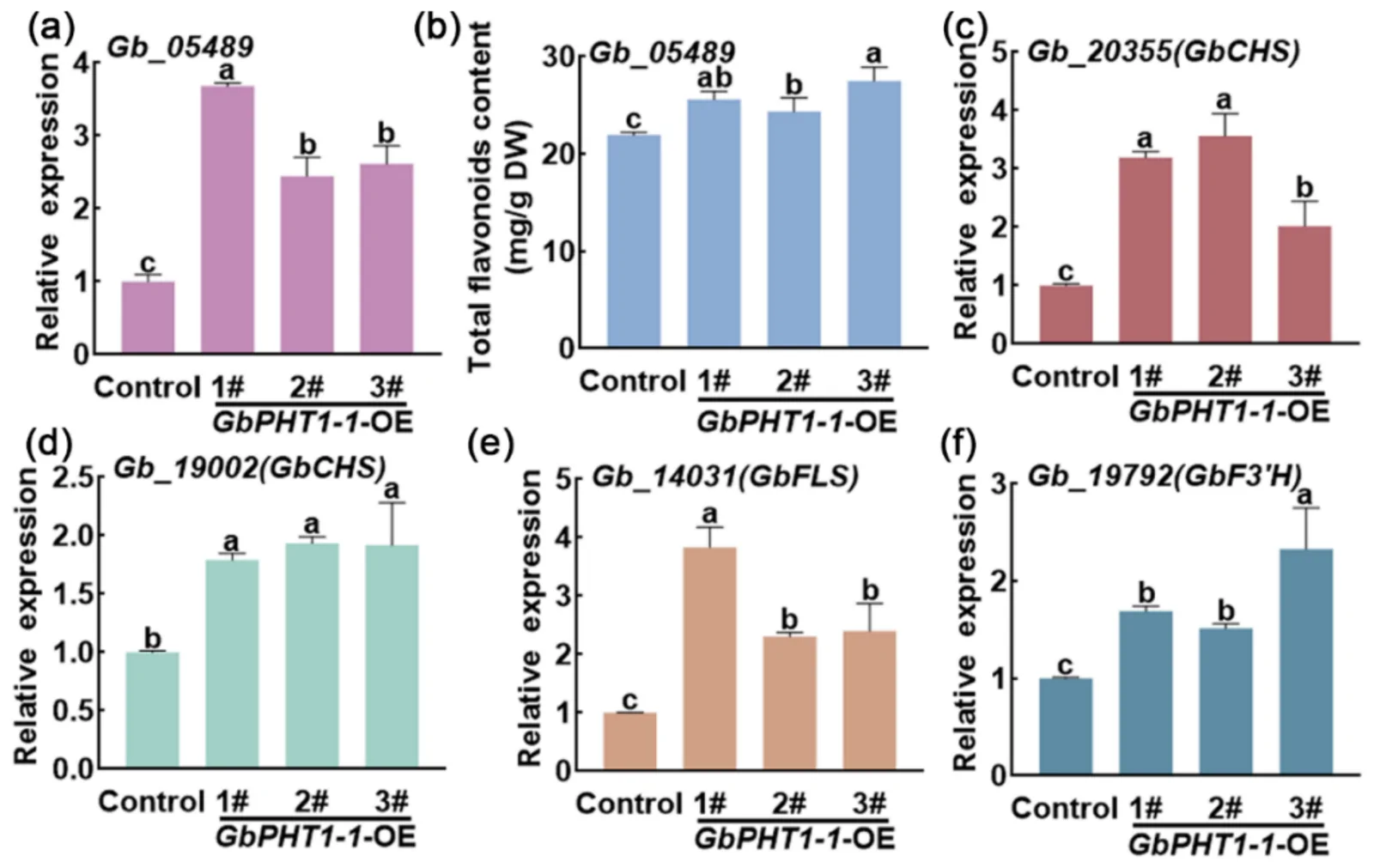
Functional characterization of *GbPHT1-1* in flavonoid accumulation through *Agrobacterium*-mediated transient overexpression in intact *G. biloba* leaves. (**a**) Relative expression level of *GbPHT1-1* after transient overexpression; (**b**) Total flavonoid content in *G. biloba* leaves transformed with the *GbPHT1-1*-OE construct or empty vector control; (**c**–**f**) Expression levels of flavonoid biosynthetic structural genes (*GbCHS*, *GbFLS*, and *GbF3′H*) after *GbPHT1-1* overexpression. Data are presented as the mean ± standard deviation (SD) of three independent biological replicates (*n* = 3). Different letters presented above the bars indicate significant differences (One-way ANOVA statistical analysis, *p* < 0.05).

**Table 1:** The physiological and biochemical properties of 21 deduced GbPHT proteins.

ID	Gene Name	aa	MW (KDa)	PI	Exon	Chr	GRAVY	Instability Index	Aliphatic Index	Subcellular Location (Prediction by CELLO)	TMHMM Server
Gb_05489	GbPHT1-1	546	60.29	8.78	1	Chr10	0.245	34.71	86.90	Plasma membrane	11
Gb_05490	GbPHT1-2	517	56.88	8.57	1	Chr10	0.256	36.39	85.16	Plasma membrane	10
Gb_07357	GbPHT1-3	544	60.00	8.72	1	Chr10	0.257	36.50	86.69	Plasma membrane	11
Gb_07356	GbPHT1-4	544	59.75	8.60	1	Chr10	0.267	33.75	87.39	Plasma membrane	11
Gb_05492	GbPHT1-5	541	59.71	8.89	1	Chr10	0.234	37.33	88.08	Plasma membrane	11
Gb_20165	GbPHT2-1	635	67.18	9.12	4	Chr03	0.430	29.95	97.26	Plasma membrane	13
Gb_16849	GbPHT3-1	328	35.64	9.22	6	Chr07	0.211	26.70	91.31	Plasma membrane	0
Gb_16858	GbPHT3-2	306	33.29	9.05	6	Chr07	0.217	28.97	88.95	Plasma membrane	0
Gb_04962	GbPHT3-3	205	22.69	9.18	3	Chr09	0.122	26.41	98.98	Plasma membrane	0
Gb_04961	GbPHT3-4	143	15.68	8.55	4	Chr09	0.330	35.37	92.03	Plasma membrane	0
Gb_40674	GbPHT4-1	115	12.11	5.94	4	Chr04	0.930	46.77	115.48	Plasma membrane	3
Gb_28293	GbPHT4-2	703	77.35	8.84	13	Chr09	0.377	36.27	98.14	Plasma membrane	10
Gb_13175	GbPHT4-3	557	60.39	9.80	8	Chr10	0.387	39.34	94.81	Plasma membrane	11
Gb_22574	GbPHT4-4	309	33.90	8.49	8	Chr10	0.394	47.45	101.62	Plasma membrane	6
Gb_13176	GbPHT4-5	552	59.24	9.45	8	Chr10	0.456	36.00	99.73	Plasma membrane	12
Gb_18639	GbPHT4-6	461	50.06	9.84	1	Chr10	0.480	37.57	102.82	Plasma membrane	12
Gb_34493	GbPHO1-1	703	82.41	9.30	16	Chr02	0.044	45.25	97.61	Plasma membrane	5
Gb_33031	GbPHO1-2	796	91.49	9.64	14	Chr03	0.127	36.52	89.56	Plasma membrane	6
Gb_36223	GbPHO1-3	139	15.96	9.91	2	Chr04	0.704	22.06	127.63	Plasma membrane	2
Gb_22129	GbPHO1-4	134	15.26	9.03	3	Chr12	0.616	21.81	117.91	Plasma membrane	0
Gb_23750	GbPHO1-5	377	44.49	8.97	8	Chr12	0.316	36.71	108.36	Plasma membrane	7

## Data Availability

The original contributions presented in this study are included in the article/Supplementary Materials. Further inquiries can be directed to the corresponding author.

## Supplementary Materials

The following supporting information can be downloaded at: https://www.mdpi.com/article/10.3390/biology15191676/s1, Table S1. Primer sequences used in the experiment.
