# Supplementary material for: Genome-Wide Characterization of Phosphate Transporters Reveals Differential Regulation of Growth and Medicinal Metabolite Accumulation Under Phosphorus Deficiency in Ginkgo biloba
Source: Biology (Basel). 2026 Sep 22;15(19):1676. doi: 10.3390/biology15191676 (PMC13663550; doi:10.3390/biology15191676)
Supplement: Supplementary file 1 [file biology-15-01676-s001.zip › biology-4517464-supplementary.pdf]

Genome-wide characterization of phosphate transporters reveals differential regulation of growth and medicinal metabolite accumulation under phosphorus deficiency in *Ginkgo biloba*

Yadi Chen[1#], Chi Zhang[1#], Fangmei Cheng[2], Qingtao Xu[1], Weixing Li[1,*]

1. College of Horticulture and Landscape Architecture, Yangzhou University, Yangzhou, Jiangsu, 225009, China;

2. Jiangsu Coastal Area Institute of Agricultural Sciences, Yancheng, Jiangsu, 224002, China

# These authors contributed equally to this work

*Corresponding author: liwx@yzu.edu.cn

Yadi Chen: yadichen@yzu.edu.cn

Chi Zhang: mz120241556@stu.yzu.edu.cn

Fangmei Cheng: chengfangmei@126.com

Qingtao Xu: mx120240866@stu.yzu.edu.cn

Supplemental Table S1 Primer sequences used in the experiment.

Gene ID	Forward primer (5'-3')	Reverse primer (5'-3')
GbPHT1-1 qPCR	GAGGAGCGTTTATTGCTGCG	TGAGTGCTTTGAGCCGCATA
GbPHT1-2 qPCR	TGCGGCTCAAAGCACTCATA	GCAGAAACCGAGGGCATTTG
GbPHT1-3 qPCR	TATTCTGGCTGGTGGTGCTG	AGCGGTTTCTGGCATCTTCA
GbPHT1-4 qPCR	CTTTCCTTTGGTCGCACAGC	AGCACCACCAGCCAGAATAC
GbPHT1-5 qPCR	CTCTACCGCCAAACGTGTCT	TTCCCATCTTGTCACCGAGC
GbPHT2-1 qPCR	TTGCCCATTGATGTGCTTGC	CACAAGTGTGTGTGTCGCAG
GbPHT3-2 qPCR	AGGTGTTCTTAGCTGTGGGC	TGTAGGCACCCATCCTCTGA
GbPHT3-3 qPCR	GTGCTCGAGAATGCAGCAAC	TGATTGTACCAACAGCGCCA
GbPHT3-4 qPCR	GGAAAGGTTGGGGAGGGAAG	AAATCTGGGCTGCGTCTGAA
GbPHT4-3 qPCR	TAGGGCACAGGCTGTTTCAG	GCGGGACTCCTGACAATTCA
GbPHT4-4 qPCR	GGGTGTTGCTATGCCTGCTA	AGACCGAGCAATACACCAGC
GbPHT4-5 qPCR	TCCCCTGCAGTTTGCATGAC	TGAGAGCTGCTACAATCGCC
GbPHT4-6 qPCR	TACAGTAGCAGCGGATGCAG	CGTTCATGAGGTGGAACCCA
GbPHO1-1 qPCR	GGGCTCTTCACAGGATGCTT	GATCCTTGCACACCTCCACA
GbPHO1-4 qPCR	GCAGACCAGCTTACCAGTCA	CTTGGTTCCATGAGCACGGT
GbPHT1-1	tccagctccaggatccATGGCCAAAAATCAATTGGAAG	gagaaagcttggatccCTACACAATTGGCATAGTTCTGGTTC
GbCHS (*Gb_19002*) qPCR	TGCAGGAGCGATGAAGGATT	CACTGCAAACCACGAGAACG
GbCHS (*Gb_20355*) qPCR	CTGCAACTGTGCTTGCCATT	GCCGTTATGTTGGGATTCGC
GbFLS (*Gb_14031*) qPCR	CGGGCGAGAGTGGAAGTTT	GGTTGTGGGCAGGTTGGATA
GbF3'H (*Gb_19792*) qPCR	TATAACTTAGCGCCTCCCCG	GCTCAACATGGAGGAGGCTT
GbAACT1 (*Gb_28733*) qPCR	GGGAGTGTGACTGCTGGAAA	AAGAGATGGCGCTGTTGTGA

Supplemental Table S1 (continued)

Gene ID	Forward primer (5’-3’)	Reverse primer (5’-3’)
GbAACT2 (*Gb_04657*) qPCR	TATGCATCGTTGGTGTCGCT	GTTGCAGGCAAGGACGAAAG
GbAACT3 (*Gb_04658*) qPCR	GAAGGAGGGAGTGTGACTGC	ACAAGGGTCATGTTGTGCCA
GbDXS1 (*Gb_34070*) qPCR	GGCCATCAGGCTTATCCTCAC	ATCAAGATTCGCTGTGGGCA
